# The Influence of Bariatric Surgery on Matrix Metalloproteinase Plasma Levels in Patients with Type 2 Diabetes Mellitus

**DOI:** 10.3390/biom14121633

**Published:** 2024-12-19

**Authors:** João Kleber de Almeida Gentile, Renato Migliore, Jaques Waisberg, Marcelo Augusto Fontenelle Ribeiro Junior

**Affiliations:** 1 Departament of Surgery, Institute of Infectology Emilio Ribas (IIER), São Paulo 01246-900, SP, Brazil; 2 Department of Surgery, Hospital São Camilo, São Paulo 02401-200, SP, Brazil; renato.migliore@hotmail.com; 3 Department of Surgery, ABC Medical School, Santo André 09060-870, SP, Brazil; jaqueswaisberg@uol.com.br; 4 Department of Surgery, R Adams Cowley Shock Trauma Center, University of Maryland, Baltimore, MD 21201, USA; drmribeiro@gmail.com

**Keywords:** bariatric surgery, morbid obesity, matrix metalloproteinases, gastric bypass, gastroplasty, diabetes mellitus

## Abstract

Background: Bariatric surgery is a safe and effective procedure for treating obesity and metabolic conditions such as type 2 *diabetes mellitus* (T2DM). Remodeling of the extracellular matrix (ECM) supports adipose tissue expansion and its metabolic activity, where matrix metalloproteinases (MMPs) play a key role in ECM regulation. The MMPs, particularly MMP-2 and MMP-9, are elevated in patients with morbid obesity, metabolic syndrome, and T2DM. Objectives: To evaluate the effect of weight loss in bariatric surgery patients using oxidative stress markers and to compare MMP levels in patients undergoing bariatric surgery. Methods: This was a prospective, controlled study including 45 morbidly obese patients with T2DM (BMI > 35 kg/m^2^) who underwent RYGB (n = 24) or VG (n = 21). Weight loss was assessed through anthropometric measurements (weight, height, BMI). MMP-2 and MMP-9 levels were measured preoperatively and at 3 and 12 months postoperatively. Results: Significant and sustained weight loss was observed after surgery in both groups, with reductions in BMI. MMP-2 and MMP-9 levels decreased significantly after one year of follow-up. Conclusions: Bariatric surgery is an effective long-term intervention for weight loss and associated comorbidities, including T2DM. MMP-2 and MMP-9 proved to be effective markers of extracellular matrix remodeling, with significant reductions following surgery.

## 1. Introduction

Type 2 diabetes mellitus (T2DM) and morbid obesity are well-known clinical conditions that continue to raise significant global health concerns [1,2,3,4]. Obesity is defined as the excessive accumulation of fat in various parts of the body or organs. It is a chronic, progressive, and relapsing disease with multifactorial causes, leading to adverse metabolic and psychosocial health consequences. One of the primary causes of obesity is an imbalance between excess stored energy and energy expenditure, which disrupts nutrient signaling and results in insufficient energy use. The evaluation of adiposity involves measuring height, weight, body mass index (BMI), waist circumference, and body fat percentage. Obesity diagnosis is primarily based on BMI thresholds, which take into account body weight and fat distribution patterns, though BMI alone is now considered insufficient for a full assessment, as obesity is a multifactorial condition.

According to data from 2017 to 2020, 42.4% of adults in the U.S. have a BMI greater than 30 kg/m^2^, while 20.9% of young people fall into the same category. Furthermore, the age-adjusted prevalence of severe obesity (BMI > 40 kg/m^2^) is 9.2% [1,2]. Currently, only around 30% of the adult population in the U.S. has a normal BMI (18–25 kg/m^2^). When race and ethnicity are considered, the highest rates of obesity are found among Black women, Native Americans, and Hispanics. It is predicted that by 2023, approximately 50% of the U.S. adult population will be obese, with 25% experiencing severe obesity [2].

In Brazil, the prevalence of obesity has risen by 72% in the last 13 years, from 11.8% in 2006 to 20.3% in 2019. It is estimated that 55.4% of the Brazilian population has a BMI over 25 kg/m^2^, with 47.1% of these being men and 53.9% women. Patients with a BMI over 30 kg/m^2^ account for 19.8% of the population [3]. By 2030, it is projected that approximately 14% of men and 20% of women globally will develop clinical obesity. Furthermore, 18% of individuals are expected to have a BMI over 30 kg/m^2^, 6% a BMI over 35 kg/m^2^, and 2% a BMI over 40 kg/m^2^ [3].

Obesity is closely associated with T2DM, a condition that has been described as a global pandemic. T2DM presents one of the greatest challenges to human health, with its rapid global spread. The prevalence of T2DM increased from 5.7% of the population in 1985–1989 to 8.7% between 2005 and 2011. In 2015, the International Diabetes Federation reported that approximately 415 million people worldwide were living with T2DM, a figure projected to rise to 642 million by 2040 [1,2,3,4]. T2DM is characterized by varying degrees of pancreatic beta-cell dysfunction and insulin resistance, and its development may also be influenced by hormonal systems such as the incretin axis [5,6]. The relationship between obesity and T2DM appears to be strongly connected to the increased concentration of MMPs, where insulin resistance is more pronounced. Although the connection between adipose tissue and the synthesis of matrix metalloproteinases (MMPs) remains unclear, a deficiency of MMPs in adipose tissue is thought to contribute to the development of T2DM [4,5]. Both diabetes mellitus (DM) and obesity are primary risk factors for cardiovascular diseases. While recent studies have highlighted the significant role of extracellular matrix metalloproteinases (MMPs) in atherosclerosis, little is known about the effects of hyperglycemia on MMP regulation in vascular cells [7].

Bariatric surgery, particularly Roux-en-Y gastric bypass (RYGB) and vertical gastrectomy (VG), is one of the most effective treatments for morbid obesity, significantly reducing weight and improving clinical comorbidities, including T2DM [5,6,7,8].

MMPs are calcium- and zinc-dependent proteases involved in extracellular matrix (ECM) synthesis, basement membrane degradation, and tissue growth factor stimulation, all of which contribute to adipogenesis and adipose tissue expansion [9,10]. 

MMPs were first identified in the early 1960s for their role in ECM protein degradation [10]. As members of the metzincin superfamily of proteases, MMPs have evolved into a family of endopeptidases called matrixins. MMPs are highly homologous multidomain metalloproteinases that break down various ECM proteins through their zinc-dependent catalytic activity. The MMP family is classified into six subgroups based on their substrate specificity and homology: collagenases (MMP-1, MMP-8), gelatinases (MMP-2, MMP-9), stromelysins (MMP-3, MMP-11), matrilysins (MMP-7, MMP-26), and membrane-type MMPs (MMP-14, MMP-15, MMP-16, MMP-17, MMP-24) [9,11,12,13].

In 2001, Bouloumié et al. first described the production of MMP-2 and MMP-9 by human adipocytes and preadipocytes, identifying them as potential regulators of adipocyte differentiation [14]. MMPs have been widely studied in other medical fields and are recognized as biomarkers for diseases related to oxidative stress, including coronary heart disease and heart failure. Plasma levels of MMPs can be detected in peripheral blood; however, the relationship between plasma and tissue levels of MMPs remains unknown and MMP levels are significantly increased in patients with T2DM and obesity [14,15,16].

It has been hypothesized that obesity and T2DM increase plasma MMP concentrations, especially gelatinases and matrilysins [17]. Therefore, bariatric surgery may reduce MMP plasma levels, and serum MMP concentrations could potentially serve as markers of surgical efficacy in controlling metabolic diseases [18,19,20]. Several studies have shown that bariatric surgery significantly reduces morbidity and mortality in obese patients and improves cardiovascular risk factors [12,15,21,22,23,24]. However, few studies have explored changes in MMP-2 and MMP-9 levels after bariatric surgery [25].

This study aims to determine plasma concentrations of MMP-2 and MMP-9, as well as clinical and laboratory parameters related to obesity throughout the follow-up period, to investigate the associations between these markers and the effects of weight loss in patients undergoing bariatric surgery.

## 2. Materials and Methods

### 2.1. Patient Recruitment and Grouping

This prospective, controlled study was conducted at São Camilo Hospital—Pompeia Unit, São Paulo, Brazil with credit to the Instituto de Assistencia Médica ao Servidor Publico de Sao Paulo (IAMSPE), São Paulo, Brazil. The study was conducted in accordance with the Declaration of Helsinki and approved by the Institutional Research Ethics Committee (CAAE: 58869422.9.0000.0062) of the São Camilo Hospital—Pompeia Unit, São Paulo/SP—Brazil in 09/08/2021. The study included adult patients of both sexes diagnosed with morbid obesity and type 2 diabetes mellitus (T2DM) who had undergone bariatric surgery. Eligible patients met the World Health Organization (WHO) diagnostic criteria for morbid obesity, defined as a BMI > 40 kg/m^2^ or BMI > 35 kg/m^2^ with comorbidities, and were aged between 18 and 65 years. Additionally, patients had to have been diagnosed with T2DM for at least six months and have undergone either Roux-en-Y gastric bypass (RYGB) or vertical gastrectomy (VG). Only patients with glycosylated hemoglobin (HbA1c) levels greater than 6.5% and who had been receiving regular follow-up care with appropriate treatment were included in the study.

Patients with a history of malignant neoplasms within the last five years, human immunodeficiency virus (HIV) infection, cardiovascular diseases, pulmonary embolism, acute or chronic renal failure, viral hepatitis, liver cirrhosis, inflammatory bowel diseases, or a history of alcohol abuse or illicit drug use were excluded.

The study evaluated patients who had undergone RYGB or VG, with weight loss assessed using anthropometric data (weight, height, and BMI). Plasma levels of matrix metalloproteinase-2 (MMP-2) and matrix metalloproteinase-9 (MMP-9) were measured at three time points: one week before surgery (M0), 3 months after surgery (M3), and 12 months postoperatively (M12). Patients were followed for 12 months and were grouped based on the surgical technique: RYGB (Group 1) or VG (Group 2). Blood samples for laboratory analysis were collected from the median cubital vein between 8:00 a.m. and 11:00 a.m., and measurements included plasma levels of MMP-2, MMP-9, glycemia, and other routine tests used in the follow-up of bariatric patients.

### 2.2. MMPs Data

Peripheral blood samples were collected in Eppendorf pressure tubes for the determination of MMP-2 and MMP-9 levels. These levels were measured using the enzyme-linked immunosorbent assay (ELISA) method, specifically the Sandwich-ELISA technique, followed by spectrophotometric analysis. The plasma/serum concentration of MMPs was determined by calculating optical density (OD) compared to the standard deviation of the ELISA kit.

The following ELISA kits were used: Human MMP-2 (Matrix Metalloproteinase 2) EL-H1445 and Human MMP-9 (Matrix Metalloproteinase 9) EL-H6075, both from ELABSCIENCE (Los Angeles, CA, USA). The ELISA was conducted using a microplate pre-coated with a specific antibody for human MMP. Standards or samples were added to the wells of the micro-ELISA plate, which combined with the antibody. A biotinylated detection antibody specific for human MMP and an Avidin–Horseradish Peroxidase (HRP) conjugate were successively added to each well, followed by incubation. After the incubation period, unbound components were washed away. A substrate reagent was then added, resulting in a blue color in wells containing human MMP, the biotinylated detection antibody, and Avidin–HRP conjugate. The enzyme–substrate reaction was halted with the addition of stop solution, turning the wells yellow. OD was measured by spectrophotometry at a wavelength of 450 nm ± 2 nm. The OD value was proportional to the concentration of human MMP. Concentrations in the samples were calculated by comparing sample OD values to a standard curve.

### 2.3. Statistical Analysis

Descriptive and inferential statistical methods were employed to analyze the sample of 45 morbidly obese diabetic patients who underwent RYGB (n = 24) or VG (n = 21). Qualitative variables were presented as absolute and relative frequency distributions, while quantitative variables were summarized using measures of central tendency and variation.

For the inferential analysis, the following statistical methods were applied:

• The Shapiro–Wilk test was used to assess the normality of quantitative variables.
• Fisher’s exact test was applied to compare the categorical variable of gender between the RYGB and VG groups.
• The Mann–Whitney U-test was used to compare quantitative variables between the RYGB and VG groups.
• Longitudinal comparisons between the time points (M0, M3, and M12) were conducted using the Friedman test.

The alpha error was set at 5% for rejecting the null hypothesis. Statistical processing was performed using Bioestat version 5.3 and SPSS version 27 (IBM, New York, NY, USA).

## 3. Results

### 3.1. Characteristics of the Study Population

Regarding gender, the groups showed a significant statistical difference (*p*-value = 0.0018 *). Regarding age, there was no statistical difference (*p*-value = 0.6409) between the groups of patients (Table 1).

### 3.2. The BMI Analysis

At baseline (M0), there was a significant difference in BMI (kg/m^2^) between the RYGB and VG groups (*p* = 0.0003). This difference remained significant at the 3-month follow-up (M3) (*p* = 0.0022). By the 12-month follow-up (M12), the difference between groups was not statistically significant (*p* = 0.0571).

**Figure 1:**
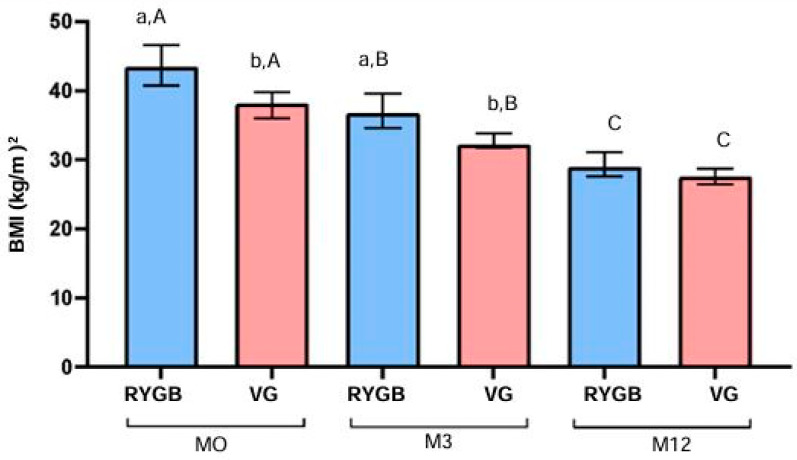
Median, 1st quartile, and 3rd quartile BMI (kg/m^2^) at M0, M3, and M12 in diabetic patients, according to surgical technique. RYGB (n = 24) and VG (n = 21). (a, b): indicate a statistically significant difference when comparing RYGB and VG at each time point, using the Mann–Whitney U-test. (A, B, C): comparison within the same group, considering the differences between M0, M3, and M12, using the Friedman test.

Within the RYGB group, BMI decreased significantly over time (*p* < 0.0001), with a reduction from a median of 41.9 kg/m^2^ at M0 to 39.5 kg/m^2^ at M3 and 28.4 kg/m^2^ at M12. This represents a 32.2% reduction in BMI after 12 months. Similarly, in the VG group, BMI also decreased significantly over time (*p* < 0.0001), with a reduction from a median of 38.2 kg/m^2^ at M0 to 32.0 kg/m^2^ at M3 and 27.6 kg/m^2^ at M12, representing a 27.7% reduction in BMI after 12 months (Figure 1).

### 3.3. MMP Analysis

MMP-2 levels did not differ significantly between the RYGB and VG groups at M0 (*p* = 0.4528), M3 (*p* = 0.7074), or M12 (*p* = 0.4949). Similarly, there were no significant differences in MMP-9 levels between the two groups at M0 (*p* = 0.2192), M3 (*p* = 0.5390), or M12 (*p* = 0.2279) (Table 2).

### 3.4. MMP-2 Analysis

**Figure 2:**
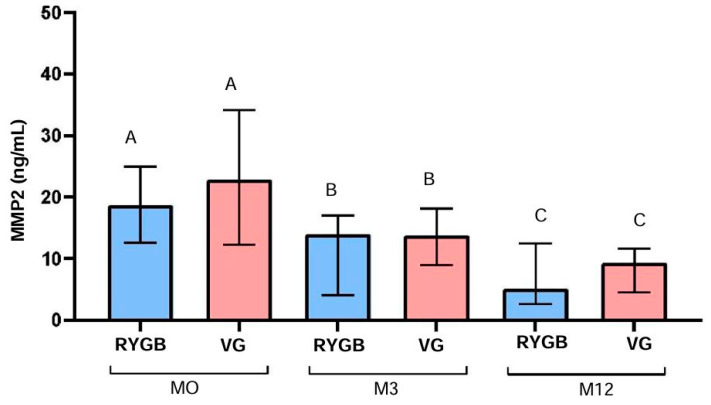
Median, 1st quartile, and 3rd quartile of MMP2 (ng/mL) at M0, M3, and M12 in diabetic patients, according to surgical technique. RYGB (n = 24) and VG (n = 21). (A, B, C): comparison within the same group, considering the differences between M0, M3, and M12, using the Friedman test.

In the RYGB group, MMP-2 levels decreased significantly over time (*p* < 0.0001), from a median of 17.7 ng/mL at M0 to 14.2 ng/mL at M3 and 5.1 ng/mL at M12. This represents a 71.2% reduction in MMP-2 levels after 12 months. Similarly, in the VG group, there was a significant reduction in MMP-2 levels over time (*p* < 0.0001), from a median of 22.9 ng/mL at M0 to 13.8 ng/mL at M3 and 9.5 ng/mL at M12, representing a 58.5% reduction in MMP-2 levels after 12 months (Figure 2).

### 3.5. MMP-9 Analysis

**Figure 3:**
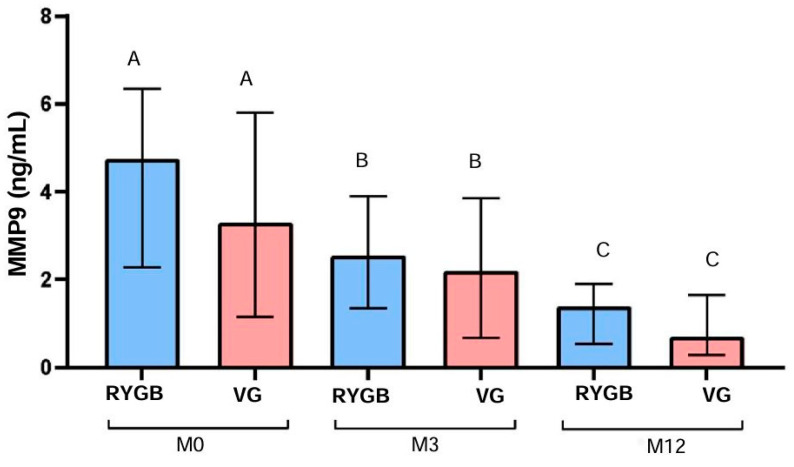
Median, 1st quartile, and 3rd quartile of MMP9 (ng/mL) at M0, M3, and M12 in diabetic patients, according to surgical technique. RYGB (n = 24) and VG (n = 21). (A, B, C): comparison within the same group, considering the differences between M0, M3, and M12, using the Friedman test.

MMP-9 levels also decreased significantly in the RYGB group over time (*p* < 0.0001), from a median of 4.7 ng/mL at M0 to 2.5 ng/mL at M3 and 1.4 ng/mL at M12, resulting in a 70.2% reduction after 12 months. In the VG group, MMP-9 levels decreased significantly as well (*p* < 0.0001), from a median of 3.3 ng/mL at M0 to 2.2 ng/mL at M3 and 0.7 ng/mL at M12, representing a 78.8% reduction after 12 months (Figure 3).

## 4. Discussion

Obesity results from the storage of lipids in adipose tissue, which leads to the accumulation of ECM, requiring the remodeling of this adipose tissue to accommodate its growth [26].

In our study, patients had successful weight loss after surgery, with a consequent reduction in BMI, proving to be an effective method for sustained weight loss. Bariatric surgery is the most effective therapeutic options for long-term weight loss and improvement of clinical comorbidities associated with obesity, such as T2DM [25]. Oxidative stress and tissue inflammation lead to pathological expansion of ECM, with aggregation of inflammatory cells (macrophages) and expression of collagen with increased accumulation of lipids. The deposition of excess ECM in adipose tissue leads to necrosis and apoptosis of adipocyte cells, with an accumulation of macrophages at the site and a consequent increase in insulin resistance [27,28].

A group of zinc-dependent endoproteases, known as MMPs, play a role in tissue remodeling and the degradation of different extracellular matrix proteins. MMPs may be involved in cell death, angiogenesis, tissue repair, and the immune response, as well as promoting cell proliferation, migration, and differentiation [27].

MMPs can modify numerous biological and signaling pathways, as well as bioactive substances on the cell surface, in addition to normal biological processes such as pregnancy, wound healing, atherosclerosis, vascular aneurysms, and alterations in MMP expression in a series of neoplasms and musculoskeletal diseases such as osteoarthritis and bone resorption [23].

Studies have shown that MMP-derived integrins can modulate glucose transport in adipose tissue, impair glucose absorption by skeletal muscle, and aggravate peripheral insulin resistance [8,29]. Integrins seem to act in the mechanical stimulation of insulin signaling through a subgroup of integrin (P2) that directly impacts insulin sensitization, leading to an imbalance in glycemia and an inflammatory process through the infiltration of leukocytes in adipose tissue, which highlights the systemic inflammatory state present in patients with any degree of obesity [8].

This mechanism related to the action of integrins was revealed by the study by Roumans et al., which demonstrated the presence of integrin genetic alterations and their association with alterations in ECM remodeling in patients with obesity. This showed that obesity is correlated with increased MMP expression in adipose tissue, with MMP-2 and MMP-9 being involved in the ECM remodeling process [30].

Current studies have shown higher levels of MMP-2 and MMP-9 in patients with obesity, metabolic syndrome, and T2DM compared to control groups [5,14,20,22]. However, one study presented different results to those obtained by Bouloumié et al., showing no significant difference between serum levels of MMP- 2 and MMP-9 in patients bearing obesity or metabolic syndrome [31].

García-Prieto et al. demonstrated MMP-2 activity that was initially similar in obese and non-obese patients, as well as greater MMP-9 activity in obese patients, which are both significant results, even though patients undergoing bariatric surgery show a decline in serum MMP-2 and MMP-9 levels. One caveat to the study is that the sample had a non-significant number of patients, which may constitute a bias in the study [32].

Compared to our study, there seems to be a positive correlation between bariatric surgery and a reduction in MMP levels, which was more evident in patients who underwent RYGB compared to VG; however, both are effective in reducing MMP levels in the long term.

Wu et al. presented the effects of bariatric surgery using the RYGB and VG techniques on the plasma concentration of MMP-2 and MMP-9. These authors showed that there was no significant difference between MMP-2 and MMP-9 values before and after patients underwent bariatric surgery, regardless of the surgical technique used. However, this study had important limitations in terms of statistical analysis, where there appears to be a type II error, as well as a patient selection bias [33].

In a 2013 prospective study, Lee YJ et al. presented a series of 37 diabetic patients undergoing bariatric surgery with a significant reduction in the concentration of MMP-2 and MMP-9. The study also compared data such as serum insulin and control of comorbidities, establishing a direct relationship between weight loss, MMP-9,and improvement in insulin resistance and control of metabolic diseases in previously obese patients [34].

Studies in rodents have shown that integrins can modulate the glucose-4 transporter in adipose tissue, impair glucose uptake in skeletal muscle, and aggravate insulin resistance [5,35].

Integrins that participate in the mechanical stimulation of insulin signaling in the localization of insulin receptors in the membrane and insulin sensitivity have an impact on glucose balance under high fat consumption, activating the immune system, which improves insulin resistance [36].

This mechanism was corroborated in a study by Roumans et al., which showed alterations in integrin gene activity and ECM remodeling in patients with obesity where therapy resulted in diet control [30].

The presence of MMPs directly affects the adhesion, migration, and differentiation of cells in adipose tissue, and their accumulation in adipose tissue results in increased insulin resistance, leading to a worsening of the glycemic profile of diabetic patients [37].

Recently, Carbone et al. discovered an integrin called osteopontin that is significantly elevated in patients with T2DM. They were able to assess that the presence of osteopontin may be related to insulin resistance and its presence may be closely related to T2DM remission in patients undergoing bariatric surgery [38].

Like other studies (Laimer et al., 2005; Derosa et al., 2008; Madsen et al., 2009), our obese patients showed a significant decrease in MMP-2 and MMP-9 activity in the post-bariatric surgery period, correlating with a decrease in BMI [17,39,40].

The influence of pro-inflammatory cytokines under the action of neutrophils induces MMP-9 activity and contributes to microvascular complications, as demonstrated by Khatri in 2002, as a marker of endothelial damage [41].

In this context, we observed a positive correlation between weight loss in obese patients and a drop in MMP-2 and MMP-9 activities, suggesting that metalloproteinases are an important marker in ECM remodeling.

This understanding may indicate that a reduction in the expression of MMP-2 and MMP-9 after bariatric surgery could be a marker of clinical improvement in obesity, since a reduction in serum MMP levels is associated with a control of extracellular matrix function in adipocytes.

However, more studies like ours are needed to confirm the association of metalloproteinases with cardiovascular risk and chronic diseases such as T2DM and to develop methods for intervention and control of metalloproteinase deposition as a treatment for chronic diseases.

## 5. Conclusions

This study demonstrates that oxidative stress markers, MMP-2 and MMP-9, significantly decrease after bariatric surgery in both RYGB and VG groups. These findings suggest that bariatric surgery is an effective intervention for controlling metabolic conditions such as T2DM. Although no significant differences were found between the two surgical techniques in terms of MMP reduction, both RYGB and VG effectively reduced MMP levels over time. This highlights the potential role of MMPs as important biomarkers for ECM remodeling and metabolic improvements post-surgery. Further studies are warranted to explore the association of MMP reduction with cardiovascular risk and chronic disease management in obese patients.

## Figures and Tables

**Table 1:** Gender and age of patients operated on using the RYGB (Group 1) or VG (Group 2) techniques.

Operative Techniques			
Features	RYGB (Group 1)	VG (Group 2)	*p*-Value
**Patients no.**	**24**	**21**	
**Sex**			0.0018 *
Male	9	0	
	37.5%	0.0%	
Female	15	21	
	62.5%	100%	
**Age**			0.6409 **
Minimum	30	23	
Maximum	67	54	
Median	40.5	43.0	
Average	41.4	40.7	
Standard Deviation	7.9	9.1	

RYGB: Roux-en-Y gastric bypass. VG: vertical gastrectomy. * *p* by Fisher’s exact test. ** *p* by Mann–Whitney U-test.

**Table 2:** BMI, MMP2, and MMP9 values in diabetic patients according to surgical technique: RYGB (n = 24) and VG (n = 21).

		BMI (kg/m^2^)			MMP-2 (ng/mL)			MMP-9 (ng/mL)		
		RYGB	VG	*p*-Value	RYGB	VG	*p*-Value	RYGB	VG	*p*-Value
**M0**	MD	41.9	38.2	0.0003 *	17.7	22.9	0.4528	4.7	3.3	0.2192
	Average	44.0	38.0		19.6	22.5		4.7	3.7	
	DP	4.9	2.2		11.0	12.6		2.7	2.7	
**M3**	MD	35.9	32.0	0.0022 *	14.1	13.8	0.7074	2.5	2.2	0.5390
	Average	29.3	27.5		12.2	13.5		2.7	2.7	
	DP	2.9	1.4		8.5	6.7		1.7	2.4	
	D(0,3)	−14.3%	−16.2%		−20.3%	−39.7%		−46.8%	−33.3%	
**M12**	MD	28.4	27.6	0.0571	5.1	9.5	0.4949	1.4	0.7	0.2279
	Average	28.8	27.5		8.1	8.5		1.4	1.3	
	DP	2.7	1.4		6.9	4.9		1.0	1.5	
	D(0,12)	−32.2%	−27.7%		−71.2%	−58.5%		−70.2%	−78.8%	
***p*****-value** (0,3,12)		<0.0001 *	<0.0001 *		<0.0001 *	<0.0001 *		<0.0001 *	<0.0001 *	

RYGB: Roux-en-Y gastric bypass. VG: vertical gastrectomy. BMI: body mass index. MMP-2: metalloproteinases-2. MMP-9: metalloproteinase-9. *p*: Mann–Whitney U-test (*p*-value), comparing the RYGB and VG groups. *p* (0,3,12): Friedman test (*p*-value), comparing the times: M0, M3 and M12. D(0,3): percentage difference between M0 and M3. D(0,12): percentage difference between M0 and M12. MD: median. SD: standard deviation.

## Data Availability

Dataset available on request from the authors.

## References

[1] GBD 2015 Obesity Collaborators (2017). Health Effects of Overweight and Obesity in 195 Countries over 25 Years. N. Engl. J. Med..

[2] Unnikrishnan R., Pradeepa R., Joshi S.R., Mohan V. (2017). Type 2 Diabetes: Demystifying the Global Epidemic. Diabetes.

[3] Morris M.J. (2008). Cardiovascular and Metabolic Effects of Obesity. Clin. Exp. Pharmacol. Physiol..

[4] Bhatt L., Addepalli V. (2015). Matrix metalloproteinases in diabesity. Diabesity.

[5] Kang J.H., Le Q.A. (2017). Effectiveness of bariatric surgical procedures. Medicine.

[6] Koliaki C., Liatis S., le Roux C.W., Kokkinos A. (2017). The role of bariatric surgery to treat diabetes: Current challenges and perspectives. BMC Endocr. Disord..

[7] Wang W., Fann C.S., Yang S.-H., Chen H.-H., Chen C.-Y. (2019). Weight loss and metabolic improvements in obese patients undergoing gastric banding and gastric banded plication: A comparison. Nutrition.

[8] Huang H.-H., Lee W.-J., Chen S.-C., Chen T.-F., Lee S.-D., Chen C.-Y. (2019). Bile Acid and Fibroblast Growth Factor 19 Regulation in Obese Diabetics, and Non-Alcoholic Fatty Liver Disease after Sleeve Gastrectomy. J. Clin. Med..

[9] Medeiros N.I., Gomes J.A.S., Fiuza J.A., Sousa G.R., Almeida E.F., Novaes R.O., Rocha V.L.S., Chaves A.T., Dutra W.O., Rocha M.O.C. (2019). MMP-2 and MMP-9 plasma levels are potential biomarkers for indeterminate and cardiac clinical forms progression in chronic Chagas disease. Sci. Rep..

[10] Visse R., Nagase H. (2003). Matrix Metalloproteinases and Tissue Inhibitors of Metalloproteinases. Circ. Res..

[11] Koh Y., Jaoude J. (2016). Matrix metalloproteinases in exercise and obesity. Vasc. Health Risk Manag..

[12] Migliore R., Gentile J.K.A., Franca F.T., Kappaz G.T., Bueno-De-Souza P.M.S., Assef J.C. (2018). Impact of Bariatric Surgery on the Inflammatory State Based on CPR Value. Arq. Bras. Cir. Dig..

[13] Rydlova M., Holubec L., Kalfert D., Franekova J., Povýšil C., Ludvíková M. (2008). Biological Activity and Clinical Implications of the Matrix Metalloproteinases. Anticancer Res..

[14] Bouloumié A., Sengenès C., Portolan G., Galitzky J., Lafontan M. (2001). Adipocyte Produces Matrix Metalloproteinases 2 and 9. Diabetes.

[15] Vandooren J., Geurts N., Martens E., Van den Steen P.E., Opdenakker G. (2013). Zymography methods for visualizing hydrolytic enzymes. Nat. Methods.

[16] Geurts N., Opdenakker G., Van den Steen P.E. (2012). Matrix metalloproteinases as therapeutic targets in protozoan parasitic infections. Pharmacol. Ther..

[17] Derosa G., Ferrari I., D’Angelo A., Tinelli C., Salvadeo S.A.T., Ciccarelli L., Piccinni M.N., Gravina A., Ramondetti F., Maffioli P. (2008). Matrix Metalloproteinase-2 and -9 Levels in Obese Patients. Endothelium.

[18] Galis Z.S., Sukhova G.K., Lark M.W., Libby P. (1994). Increased expression of matrix metalloproteinases and matrix degrading activity in vulnerable regions of human atherosclerotic plaques. J. Clin. Investig..

[19] Thorp E.B. (2012). Contrasting Inflammation Resolution during Atherosclerosis and Post Myocardial Infarction at the Level of Monocyte/Macrophage Phagocytic Clearance. Front. Immunol..

[20] Hopps E., Presti R.L., Montana M., Noto D., Averna M.R., Caimi G. (2013). Gelatinases and Their Tissue Inhibitors in a Group of Subjects with Metabolic Syndrome. J. Investig. Med..

[21] Ganji S.H., Kamanna V.S., Kashyap M.L. (2014). Niacin decreases leukocyte myeloperoxidase: ECMhanistic role of redox agents and Src/p38MAP kinase. Atherosclerosis.

[22] Anatoliotakis N., Deftereos S., Bouras G., Giannopoulos G., Tsounis D., Angelidis C., Kaoukis A., Stefanadis C. (2013). Myeloperoxidase: Expressing Inflammation and Oxidative Stress in Cardiovascular Disease. Curr. Top. Med. Chem..

[23] Pulli B., Ali M., Forghani R., Schob S., Hsieh K.L.C., Wojtkiewicz G., Linnoila J.J., Chen J.W. (2013). Measuring Myeloperoxidase Activity in Biological Samples. PLoS ONE.

[24] Mayyas F.A., Al-Jarrah M.I., Ibrahim K.S., Alzoubi K.H. (2014). Level and significance of plasma myeloperoxidase and the neutrophil to lymphocyte ratio in patients with coronary artery disease. Exp. Ther. Med..

[25] Rao S.R. (2012). Inflammatory markers and bariatric surgery: A meta-analysis. Inflamm. Res..

[26] Hammarstedt A., Gogg S., Hedjazifar S., Nerstedt A., Smith U. (2018). Impaired Adipogenesis and Dysfunctional Adipose Tissue in Human Hypertrophic Obesity. Physiol. Rev..

[27] Strissel K.J., Stancheva Z., Miyoshi H., Perfield J.W., DeFuria J., Jick Z., Greenberg A.S., Obin M.S. (2007). Adipocyte death, adipose tissue remodeling, and obesity complications. Diabetes.

[28] Bobulescu I.A., Lotan Y., Zhang J., Rosenthal T.R., Rogers J.T., Adams-Huet B., Sakhaee K., Moe O.W. (2014). Triglycerides in the Human Kidney Cortex: Relationship with Body Size. PLoS ONE.

[29] Kang L., Ayala J.E., Lee-Young R.S., Zhang Z., James F.D., Neufer P.D., Pozzi A., Zutter M.M., Wasserman D.H. (2011). Diet-induced muscle insulin resistance is associated with extracellular matrix remodeling and interaction with integrin α_2_β_1_ in mice. Diabetes..

[30] Roumans N.J., Vink R.G., Fazelzadeh P., van Baak M.A., Mariman E.C. (2017). A role for leukocyte integrins and extracellular matrix remodeling of adipose tissue in the risk of weight regain after weight loss. Am. J. Clin. Nutr..

[31] Papazafiropoulou A., Perrea D., Moyssakis I., Kokkinos A., Katsilambros N., Tentolouris N. (2010). Plasma levels of MMP-2, MMP-9 and TIMP-1 are not associated with arterial stiffness in subjects with type 2 diabetes mellitus. J. Diabetes Its Complicat..

[32] García-Prieto C.F., Gil-Ortega M., Vega-Martín E., Ramiro-Cortijo D., Martín-Ramos M., Bordiú E., Sanchez-Pernaute A., Torres A., Aránguez I., Fernández-Alfonso M. (2019). Beneficial Effect of Bariatric Surgery on Abnormal MMP-9 and AMPK Activities: Potential Markers of Obesity-Related CV Risk. Front. Physiol..

[33] Wu W.-C., Lee W.-J., Lee T.-H., Chen S.-C., Chen C.-Y. (2020). Do different bariatric surgical procedures influence plasma levels of matrix metalloproteinase-2, -7, and -9 among patients with type 2 diabetes mellitus?. World J. Diabetes.

[34] Lee Y.J., Heo Y.-S., Park H.S., Lee S.H., Lee S.K., Jang Y.J. (2013). Serum SPARC and Matrix Metalloproteinase-2 and Metalloproteinase-9 Concentrations after Bariatric Surgery in Obese Adults. Obes. Surg..

[35] Zong H., Bastie C.C., Xu J., Fassler R., Campbell K.P., Kurland I.J., Pessin J.E. (2009). Insulin Resistance in Striated Muscle-specific Integrin Receptor β1-deficient Mice. J. Biol. Chem..

[36] Meakin P.J., Morrison V.L., Sneddon C.C., Savinko T., Uotila L., Jalicy S.M., Gabriel J.L., Kang L., Ashford M.L.J., Fagerholm S.C. (2015). Mice Lacking beta2-Integrin Function Remain Glucose Tolerant in Spite of Insulin Resistance, Neutrophil Infiltration and Inflammation. PLoS ONE.

[37] McCulloch L.J., Rawling T.J., Sjöholm K., Franck N., Dankel S.N., Price E.J., Knight B., Liversedge N.H., Mellgren G., Nystrom F. (2015). COL6A3 Is Regulated by Leptin in Human Adipose Tissue and Reduced in Obesity. Endocrinology.

[38] Carbone F., Adami G., Liberale L., Bonaventura A., Bertolotto M., Andraghetti G., Scopinaro N., Camerini G.B., Papadia F.S., Cordera R. (2019). Serum levels of osteopontin predict diabetes remission after bariatric surgery. Diabetes Metab..

[39] Laimer M., Kaser S., Kranebitter M., Sandhofer A., Mühlmann G., Schwelberger H., Weiss H., Patsch J.R., Ebenbichler C.F. (2005). Effect of pronounced weight loss on the nontraditional cardiovascular risk marker matrix metalloproteinase-9 in middle-aged morbidly obese women. Int. J. Obes..

[40] Madsen E.L., Bruun J.M., Skogstrand K., Hougaard D.M., Christiansen T., Richelsen B. (2009). Long-term weight loss decreases the nontraditional cardiovascular risk factors interleukin-18 and matrix metalloproteinase-9 in obese subjects. Metabolism.

[41] Galis Z.S., Khatri J.J. (2002). Matrix Metalloproteinases in Vascular Remodeling and Atherogenesis. Circ. Res..

